# Complexity Analysis and Parameter Estimation of Dynamic Metabolic Systems

**DOI:** 10.1155/2013/698341

**Published:** 2013-10-23

**Authors:** Li-Ping Tian, Zhong-Ke Shi, Fang-Xiang Wu

**Affiliations:** ^1^School of Information, Beijing Wuzi University, Beijing 101149, China; ^2^School of Atuomation, Northwestern Polytechnical University, Xi'an, Shaanxi 710072, China; ^3^Department of Mechanical Engineering, University of Saskatchewan, 57 Campus Drive, Saskatoon, SK, Canada S7N 5A9; ^4^Division of Biomedical Engineering, University of Saskatchewan, 57 Campus Drive, Saskatoon, SK, Canada S7N 5A9

## Abstract

A metabolic system consists of a number of reactions transforming molecules of one kind into another to provide the energy that living cells need. Based on the biochemical reaction principles, dynamic metabolic systems can be modeled by a group of coupled differential equations which consists of parameters, states (concentration of molecules involved), and reaction rates. Reaction rates are typically either polynomials or rational functions in states and constant parameters. As a result, dynamic metabolic systems are a group of differential equations nonlinear and coupled in both parameters and states. Therefore, it is challenging to estimate parameters in complex dynamic metabolic systems. In this paper, we propose a method to analyze the complexity of dynamic metabolic systems for parameter estimation. As a result, the estimation of parameters in dynamic metabolic systems is reduced to the estimation of parameters in a group of decoupled rational functions plus polynomials (which we call improper rational functions) or in polynomials. Furthermore, by taking its special structure of improper rational functions, we develop an efficient algorithm to estimate parameters in improper rational functions. The proposed method is applied to the estimation of parameters in a dynamic metabolic system. The simulation results show the superior performance of the proposed method.

## 1. Introduction

Living cells require energy and material for maintaining their essential biological processes through metabolism, which is a highly organized process. Metabolic systems are defined by the enzymes dynamically converting molecules of one type into molecules of another type in a reversible or irreversible manner. Modeling and parameter estimation in dynamic metabolic systems provide new approaches towards the analysis of experimental data and properties of the systems, ultimately leading to a great understanding of the language of living cells and organisms. Moreover, these approaches can also provide systematic strategies for key issues in medicine, pharmaceutical, and biotechnological industries [1]. The formulation and identification of metabolic systems generally includes the building of the mathematical model of biological process and the estimating of system parameters. Because the components of a pathway interact not only with each other in the same pathway but also with those in different pathways, most (if not all) of mathematical models of metabolic systems are highly complex and nonlinear. The widely used approaches for modeling inter- and intracellular dynamic processes are based on mass action law [1–4]. By mass action law, the reaction rates are generally polynomials in concentrations of metabolites with reaction constants or rational functions which are a fraction and whose denominator and numerators are polynomials in concentrations of metabolites with reaction constants [1–4]. As a result, the mathematical model is nonlinear not only in the states but also in the parameters. Estimation of these parameters is crucial to construct a whole metabolic system [5–7].

In general, all algorithms for nonlinear parameter estimation can be used to estimate parameters in metabolic systems, for example, Gauss-Newton iteration method, and its variants such as Box-Kanemasu interpolation method, Levenberg damped least squares methods and Marquardt's method [8, 9]. However, these iteration methods are initial-sensitive. Another main shortcoming is that these methods may converge to the local minimum of the least squares cost function and thus cannot find the real values of parameters. Furthermore, because of their highly complexity and nonlinearity, Gauss-Newton iteration method and its variants cannot efficiently and accurately estimate the parameters in metabolic systems [5–7, 10, 11].

 In this paper, we propose a systematic method for estimating parameters in dynamic metabolic systems. Typically mathematical model of dynamic metabolic systems consists of a group of nonlinear differential equations, some of which contains several rational functions in which parameters are nonlinear. In Section 2, we propose a method for model complexity analysis via the stoichiometric matrix. As a result, we obtain a group of equations, each of which contains only one-rational function plus polynomial functions which we called an improper rational function. Then, based on the observation that in the improper rational functions both the denominator and numerator are linear in parameters while polynomials are also linear in parameters, we develop an iterative linear least squares method for estimating parameters in dynamic metabolic systems in Section 3. The basic idea is to transfer optimizing a nonlinear least squares objective function into iteratively solving a sequence of linear least squares problems. In Section 4, we apply our developed method to estimate parameters in a metabolism system. Finally we give conclusions and some directions of future work along with this study in Section 5.

## 2. Model Complexity Analysis for Parameter Estimation

A dynamic metabolic system consists of *k* substances (molecules), and *m* reactions can be described by a system of differential equations as follows:

(1) $$\begin{array}{c} \frac{dx_{i}}{dt}=\sum_{j=1}^{m}c_{ij}r_{j},\;\text{for}\;\;i=1,\ldots ,k, \end{array}$$

where *x*_*i*_ represents the concentrations of molecule *i*, *r*_*j*_ represents the reaction rate *j*, and *c*_*ij*_ represents the stoichiometric coefficient of molecule *i* in reaction *j*. The mass action law in biochemical kinetics [2–4, 12] states that the reaction rate is proportional to the probability of a collision of the reactants. This probability is in turn proportional to the concentration of reactants. Therefore, reaction rate *r*_*j*_ is a function of the concentrations of molecules involved in reaction *j* and proportion constants. 

The stoichiometric coefficient *c*_*ij*_ assigned to molecule *i* and reaction *j* can be put into a so-called stoichiometric matrix **C** = [*c*_*ij*_]_*k*×*m*_. Let *X* = [*x*_1_, *x*_2_,…, *x*_*k*_]^*T*^ and **r** = [*r*_1_, *r*_2_,…, *r*_*m*_]^*T*^, and let **β** = [*β*_1_,*β*_2_,…,*β*_*p*_]^*T*^ represent the vector consisting of all independent proportion constants, and then (1) can be rewritten in the following vector-matrix format:

(2) $$\begin{array}{c} \frac{dX}{dt}=Cr\left(X,\beta \right). \end{array}$$

In principle, the stoichiometric coefficient *c*_*ij*_ in matrix **C** is a constant integer and can be decided according to how molecule *i* is involved in reaction *j*. According to mass action law, the expression of reaction rates can be determined to be polynomials or rational functions with reaction constants [2–4, 12]. The challenge to build up the mathematic model of dynamic metabolic system (2) is to estimate the parameter vector **β**, especially when some reaction rates are in the form of rational functions in which parameters are nonlinear. 

If each differential equation in (2) contains one-rational function without or with polynomial functions, the parameters in model (2) can be estimated by algorithms in [13, 14] or a new algorithm proposed in the next section of this paper. Unfortunately, each differential equation contains a linear combination of several rational functions, which makes the parameter estimation in those coupled differential equations more difficult. The stoichiometric matrix contains very important information about the structure of the metabolic systems and is widely used to analyze the steady state and flux balance of metabolic systems [2–4]. In this paper, via the stoichiometric matrix, we propose a systematic method to transfer a system of differential equations (2) into another system of differential equations, in which each differential equation contains at most one-rational function.

**Figure 1:**
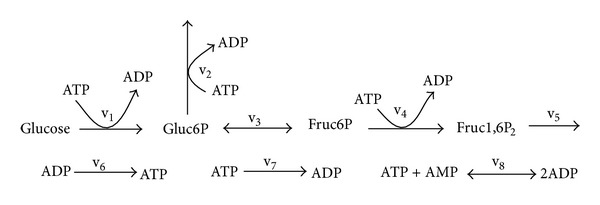
Schematic representation of the upper part of glycolysis [4].

*Running Example*. To illustrate the proposed method, we use the upper part of glycolysis system as a running example, showing how the method is applied to this system step after step. The schematic representation of this system is shown in Figure 1. The model for this metabolic system is described by the system of differential equations (2) as follows:

(3) $$\begin{array}{c} \frac{d}{dt}\text{Gluc}6\text{P}=r_{1}-r_{2}-r_{3},\\ \frac{d}{dt}\text{Fruc}6\text{P}=r_{3}-r_{4},\\ \frac{d}{dt}\text{Fruc}1,6{\text{P}}_{2}=r_{4}-r_{5},\\ \frac{d}{dt}\text{ATP}=-r_{1}-r_{2}-r_{4}+r_{6}-r_{7}-r_{8},\\ \frac{d}{dt}\text{ADP}=r_{1}+r_{2}+r_{4}-r_{6}+r_{7}+2r_{8},\\ \frac{d}{dt}\text{AMP}=-r_{8}. \end{array}$$

Based on the mass action law, the individual reaction rates can be expressed as

(4) r1=Vmax⁡,2ATP(t)KATP,1+ATP(t),r2=k2ATP(t)·Gluc6P(t),r3=(Vmax⁡,3fKGluc6P,3Gluc6P(t)    −Vmax⁡,3rKFruc6P,3Fruc6P(t))  ×(1+(Gluc6P(t)KGluc6P,3)     +Fruc6P(t)KFruc6P,3)−1,r4=Vmax⁡,4(Fruc6P(t))2KFruc6P,4(1+κ(ATP(t)/AMP(t))2)+(Fruc6P(t))2,r5=k5Fruc1,6P2(t),r6=k6ADP(t),r7=k7ATP(t),r8=k8fATP(t)·AMP(t)−k8r(ADP(t))2.

Model (3) has six ordinary differential equations (ODEs) and 15 parameters contained in eight reaction rates, three out of which are rational functions. Some ODEs contain more than one rational reaction rates, which makes the parameter more difficult.

Comparing (3) to (2) we have the state vector: **X** = [Gluc6P; Fruc6P; Fruc1,6P_2_; ATP, ADP, AMP] and stoichiometric matrix:

(5) C=[1−1−100000001−100000001−1000−1−10−101−1−111010−1120000000−1].

In the following, we describe our proposed method to analyze the complexity of model (2) through the running example.

**Step 1**

Collect the columns in the stoichiometric matrix corresponding to the rational reaction rates in model (2) to construct a submatrix **C**_*r*_ and collect other columns (corresponding to polynomial reaction rates) to construct a submatrix **C**_*p*_. Therefore, we have

(6) $$\begin{array}{c} \frac{dX}{dt}=Cr\left(X,\beta \right)=C_{r}r_{r}\left(X,\beta \right)+C_{p}r_{p}\left(X,\beta \right), \end{array}$$

where **r**_*r*_ is the subvector of **r** and consists of all rational reaction rates while **r**_*p*_ is another subvector of **r** and consists of all polynomial reaction rates. In this step, we should make sure that the rank of matrix **C**_*r*_ equals the number of rational reaction rates. If the rank of matrix **C**_*r*_ does not equal the number of rational reaction rates, it means that some rational reaction rates are not independent. Then we combine dependent rational reaction rates together to create a new reaction rate such that all resulted rational reaction rates should be linearly independent [14]. As a result, the rank of matrix **C**_*r*_ will equal the number of rational reaction rates.

For the running example, we have

(7) Cr=[c1,c3,c4]=[1−1001−1001−10−1101000],Cp=[c2,c5,c6,c7,c8]=[−10000000000−1000−101−1−110−1120000−1],

and **r**_*r*_ = [*r*_1_, *r*_3_, *r*_4_] and **r**_*p*_ = [*r*_2_, *r*_5_, *r*_6_, *r*_7_, *r*_8_]. The rank of matrix **C**_*r*_ equals 3, which is the number of rational reaction rates.

**Step 2**

Calculate the left inverse matrix of **C**_*r*_. That is, calculate **C**_*r*_^−^ such that

(8) $$\begin{array}{c} C_{r}^{-}C_{r}=I. \end{array}$$

As matrix **C**_*r*_ has the column full rank, matrix **C**_*r*_^−^ satisfying (8) exists although it is typically not unique. For a given matrix **C**_*r*_, **C**_*r*_^−^ can be easily found by solving (8) which is a linear algebraic system. If it is not unique, any matrix satisfying (8) works for our proposed method.

For the running example, we can have

(9) $$\begin{array}{c} C_{r}^{-}=\left[\begin{array}{cccccc} 1 & 1 & 1 & 0 & 0 & 0\\ 0 & 1 & 1 & 0 & 0 & 0\\ 0 & 0 & 1 & 0 & 0 & 0 \end{array}\right]. \end{array}$$

**Step**

Multiply (6) by matrix **C**_*r*_^−^ from the left to obtain

(10) Cr−dXdt=Cr−Crrr(X,β)+Cr−Cprp(X,β)=rr(X,β)+Cr−Cprp(X,β)

or

(11) $$\begin{array}{c} r_{r}\left(X,\beta \right)+C_{r}^{-}C_{p}r_{p}\left(X,\beta \right)=C_{r}^{-}\frac{dX}{dt}. \end{array}$$

From its expression, each differential equation in the system (11) contains only one-rational reaction rates plus a linear combination of polynomial reaction rates.

For the running example, we have

(12) $$\begin{array}{c} r_{1}-r_{2}-r_{5}=\frac{d}{dt}\left(\text{Gluc}6\text{P}+\text{Fruc}6\text{P}+\text{Fruc}1,6{\text{P}}_{2}\right),\\ r_{3}-r_{5}=\frac{d}{dt}\left(\text{Fruc}6\text{P}+\text{Fruc}1,6{\text{P}}_{2}\right),\\ r_{4}-r_{5}=\frac{d}{dt}\text{Fruc}1,6P_{2}. \end{array}$$

**Step 4**

Calculate matrix **C**_*r*_^⊥^ such that

(13) $$\begin{array}{c} C_{r}^{⊥}C_{r}=0, \end{array}$$

where **C**_*r*_^⊥^ has the full row rank and rank⁡(**C**_*r*_^⊥^) + rank⁡(**C**_*r*_^−^) = the  number  of  rows  in  **C**_*r*_. Note that **C**_*r*_^⊥^ can be easily found by solving (13), which is a homogenous linear algebraic system. Again if it is not unique, any matrix satisfying (13) works for our proposed method.

Then multiply (6) by matrix **C**_*r*_^⊥^ from the left to obtain

(14) $$\begin{array}{c} C_{r}^{⊥}\frac{dX}{dt}=C_{r}^{⊥}C_{r}r_{r}\left(X,\beta \right)+C_{r}^{⊥}C_{p}r_{p}\left(X,\beta \right)=C_{r}^{⊥}C_{p}r_{p}\left(X,\beta \right) \end{array}$$

or

(15) $$\begin{array}{c} C_{r}^{⊥}C_{p}r_{p}\left(X,\beta \right)=C_{r}^{⊥}\frac{dX}{dt}. \end{array}$$

For the running example, we can have

(16) Cr⊥=[112100000110000001],Cr⊥Cp=[−2−21−1−1000010000−1].

**Step 5**

Let *D* = **C**_*r*_^⊥^ **C**_*p*_. If rank⁡(*D*) ≥ the  number  of  columns, then solving (15) yields

(17) $$\begin{array}{c} r_{p}\left(X,\beta \right)={\left(D^{T}D\right)}^{-1}D^{T}C_{r}^{⊥}\frac{dX}{dt}. \end{array}$$

If rank⁡(*D*) < the  number  of  columns, it means that some polynomial reaction rates in (15) are linearly dependent. Then combine the linearly dependent rates and construct a new reaction rate vector ${\overset{-}{r}}_{p}(X,\beta )$ and full column rank matrix $\overset{-}{D}$ such that

(18) $$\begin{array}{c} \overset{-}{D}{\overset{-}{r}}_{p}\left(X,\beta \right)=Dr_{p}\left(X,\beta \right)=C_{r}^{⊥}C_{p}r_{p}\left(X,\beta \right)=C_{r}^{⊥}\frac{dX}{dt}, \end{array}$$

and then solving (18) yields

(19) $$\begin{array}{c} {\overset{-}{r}}_{p}\left(X,\beta \right)=\left({\overset{-}{D}}^{T}\overset{-}{D}\right){\overset{-}{D}}^{T}C_{r}^{⊥}\frac{dX}{dt}. \end{array}$$

For the running example, we have rank⁡(*D*) < the  number  of  columns. As the first four columns are linearly dependent, we can have a new reaction rates −2*r*_2_ − 2*r*_5_ + *r*_6_ − *r*_7_. Therefore, we have

(20) D−=[1−1010−1],  D−TCr⊥=[112100−1−1−201−1],

and furthermore, noting that (*d*/*dt*)(ATP + ADP + AMP) = 0, from (19) we have

(21) r6−r7−2r2−2r5 =ddt(Gluc6P+Fruc6P      + 2Fruc1,6P2+ATP−AMP),r8=−ddtAMP.

After these five steps, dynamic metabolic system (2) is transferred into a system of differential equations, in which each differential equation contains one-rational function plus polynomial functions ((11) or (12)) or only polynomial function ((19) or (21)). Parameters in (19) can be analytically estimated by well-known least squares methods. In the next section, we describe an algorithm to estimate parameters in (11). 

## 3. Parameter Estimation Algorithm

 After its complexity analysis, estimating parameters in dynamic metabolic system is reduced to mainly estimating parameters in a rational function plus polynomial, which we call the improper rational function. These functions are nonlinear in both parameters and state variables. Therefore, estimation of parameters in these models is a nonlinear estimation problem. In general, all algorithms for nonlinear parameter estimation can be used to estimate parameters in the improper rational functions, for example, Gauss-Newton iteration method and its variants such as Box-Kanemasu interpolation method, Levenberg damped least squares methods, Marquardt's method [9–12, 15], and more sophisticated methods [16]. However, these iteration methods are initial sensitive. Another main shortcoming is that most of these methods may converge to the local minimum of the least squares cost function and thus cannot find the real values of parameters. In the following, we describe an iterative linear least squares method to estimate parameters in the improper rational functions. The basic idea is to transfer optimizing a nonlinear least squares objective function into iteratively solving a sequence of linear least squares problems.

Consider the general form of the following improper rational functions:

(22) $$\begin{array}{c} \eta \left(X,\beta \right)=\frac{N_{0}\left(X\right)+\sum_{i=1}^{p_{N}}N_{i}\left(X\right)\beta_{N_{i}}}{D_{0}\left(X\right)+\sum_{j=1}^{p_{D}}D_{j}\left(X\right)\beta_{D_{j}}}+\sum_{k=1}^{p_{P}}P_{k}\left(X\right)\beta_{P_{k}}, \end{array}$$

where the vector **X** consists of the state variables and the *p*-dimensional vector **β** consists of all parameters in the improper rational function (22), which can naturally be divided into three groups: those in the numerator of the rational functions *β*_*N*_*i*__  (*i* = 1,…, *p*_*N*_), those in the denominator of the rational function *β*_*D*_*j*__  (*j* = 1,…, *p*_*D*_), and those in the polynomial *β*_*P*_*k*__  (*k* = 1,…, *p*_*P*_), where we have that *p*_*D*_ + *p*_*N*_ + *p*_*P*_ = *p*. *N*_*i*_(**X**)  (*i* = 0,1,…, *p*_*N*_), *D*_*j*_(**X**)  (*j* = 0,1,…, *p*_*D*_), and *P*_*k*_(**X**)  (*k* = 1,…, *p*_*P*_) are the known functions nonlinear in the state variable **X** and do not contain any unknown parameters. Either *N*_0_(**X**) or *D*_0_(**X**) must be nonzero, and otherwise from sensitivity analysis [9, 16] the parameters in model (22) cannot be uniquely identified.

If there is no polynomial part, model (22) is reduced to a rational function. Recently, several methods have been proposed for estimating parameters in rational functions [5, 6, 13, 14]. The authors in [5, 6] have employed general nonlinear parameter estimation methods to estimate parameters in rational functions. As shown in their results, the estimation error is fairly large. We have observed that in rational functions both the denominator and numerator are linear in the parameters. Based on this observation, we have developed iterative linear least squares methods in [13, 14] for estimating parameters in rational functions. Mathematically, improper rational function (22) can be rewritten as the following rational function:

(23) η(X,β)=(N0(X)+∑i=1pNNi(X)βNi+(∑k=1pPPk(X)βPk)   ×(D0(X)+∑j=1pDDj(X)βDj)) ×(D0(X)+∑j=1pDDj(X)βDj)−1.

However, in the numerator of the model above, there are *p*_*D*_ *p*_*P*_ + *p*_*N*_ + *p*_*P*_ coefficients while there are *p*_*D*_ + *p*_*N*_ + *p*_*P*_ unknown parameters. When *p*_*P*_ = 1, the number of parameters is equal to the numbers of coefficients, and the methods developed in [13, 14] can be applied. However, when *p*_*P*_ > 1, those methods are not applicable as the number of parameters is less than the number of coefficients in the numerator.

 In order to describe an algorithm to estimate parameters in the improper rational function (22) for *n* given groups of observation data *y*_*t*_ and **X**_*t*_  (*t* = 1,2,…, *n*), we introduce the following notation:

(24) $$\begin{array}{c} \beta_{N}={\left[\beta_{N1},\beta_{N2},\ldots ,\beta_{Np_{N}}\right]}^{T}\in R^{p_{N}},\\ \beta_{D}={\left[\beta_{D1},\beta_{D2},\ldots ,\beta_{Dp_{D}}\right]}^{T}\in R^{p_{D}},\\ \beta_{P}={\left[\beta_{P1},\beta_{P2},\ldots ,\beta_{Pp_{D}}\right]}^{T}\in R^{p_{P}},\\ \beta ={\left[\begin{array}{cc} \begin{array}{cc} \beta_{P}^{T} & \beta_{N}^{T} \end{array} & \beta_{D}^{T} \end{array}\right]}^{T},\\ \varphi_{N}\left(X_{t}\right)=\left[N_{1}\left(X_{t}\right),N_{2}\left(X_{t}\right),\ldots ,N_{p_{N}}\left(X_{t}\right)\right]\in R^{p_{N}},\\ \varphi_{D}\left(X_{t}\right)=\left[D_{1}\left(X_{t}\right),D_{2}\left(X_{t}\right),\ldots ,D_{p_{D}}\left(X_{t}\right)\right]\in R^{p_{D}},\\ \varphi_{P}\left(X_{t}\right)=\left[P_{1}\left(X_{t}\right),P_{2}\left(X_{t}\right),\ldots ,P_{p_{P}}\left(X_{t}\right)\right]\in R^{p_{P}},\\ Y={\left[y(1),y(2),\ldots ,y(n)\right]}^{T}\in R^{n},\\ \Phi_{N_{0}}={\left[N_{0}\left(X_{1}\right),N_{0}\left(X_{2}\right),\ldots ,N_{0}\left(X_{n}\right)\right]}^{T}\in R^{n},\\ \Phi_{D_{0}}={\left[D_{0}(X_{1}),D_{0}(X_{2}),\ldots ,D_{0}(X_{n})\right]}^{T}\in R^{n},\\ \Phi_{N}=\left[\begin{array}{c} \varphi_{N}\left(X_{1}\right)\\ \varphi_{N}\left(X_{2}\right)\\ \vdots \\ \varphi_{N}\left(X_{n}\right) \end{array}\right]\in R^{n\times p_{N}},\\ \Phi_{D}=\left[\begin{array}{c} \varphi_{D}\left(X_{1}\right)\\ \varphi_{D}\left(X_{2}\right)\\ \vdots \\ \varphi_{D}\left(X_{n}\right) \end{array}\right]\in R^{n\times p_{D}},\\ \Phi_{P}=\left[\begin{array}{c} \varphi_{P}\left(X_{1}\right)\\ \varphi_{P}\left(X_{2}\right)\\ \vdots \\ \varphi_{P}\left(X_{n}\right) \end{array}\right]\in R^{n\times p_{P}},\\ \Psi \left(\beta_{D}\right)=\mathrm{diag}\left[\begin{array}{c} D_{0}\left(X_{1}\right)+\varphi_{D}\left(X_{1}\right)\beta_{D}\\ D_{0}\left(X_{2}\right)+\varphi_{D}\left(X_{2}\right)\beta_{D}\\ \vdots \\ D_{0}\left(X_{n}\right)+\varphi_{D}\left(X_{n}\right)\beta_{D} \end{array}\right]\in R^{n\times n}. \end{array}$$

To estimate parameters in the improper rational function (22), as in [11], we form a sum of the weighted squared errors (the cost function) with the notions above as follows:

(25) J(β)=J(βP,βN,βD)=∑(D0(Xt)+φD(Xt)βD)2 ×(N0(Xt)+φN(Xt)βND0(Xt)+φD(Xt)βD+ΦPβP−yt)2.

Minimizing *J*(**β**) with respect to **β** = [**β**_*P*_^*T*^,**β**_*N*_^*T*^,**β**_*D*_^*T*^]^*T*^ can give the nonlinear least squares estimation of parameters **β**_*P*_, **β**_*N*_, and **β**_*D*_. We rewrite the objective function (22) as follows:

(26) J(β)=∑[(D0(Xt)+φD(Xt)βD)ΦPβP+φN(Xt)βN    −φD(Xt)ytβD−D0(Xt)yt+N0(Xt)]2.

In the objective function (26), for a given parameters ${\overset{-}{\beta }}_{D}$ in the first term, we have

(27) J(β)=J(βP,βN,βD,β−D)=[A(β−D)β−b]T[A(β−D)β−b],

where

(28) $$\begin{array}{c} A\left({\overset{-}{\beta }}_{D}\right)=\left[\begin{array}{c} \Psi \left({\overset{-}{\beta }}_{D}\right)\Phi_{P}^{T}\\ \Phi_{N}^{T}\\ -\mathrm{diag}\left(Y\right)\Phi_{D}^{T} \end{array}\right]\in R^{n\times p}, \end{array}$$

(29) $$\begin{array}{c} b=\left(\Phi_{D_{0}}\mathrm{diag}\left(Y\right)-\Phi_{N_{0}}\right)\in R^{n}. \end{array}$$

Then for given parameters ${\overset{-}{\beta }}_{D}$, we can estimate the parameters **β** = [**β**_*P*_^*T*^,**β**_*N*_^*T*^,**β**_*D*_^*T*^]^*T*^ by linear least squares method as follows:

(30) $$\begin{array}{c} \beta ={\left[A^{T}\left({\overset{-}{\beta }}_{D}\right)A\left({\overset{-}{\beta }}_{D}\right)\right]}^{-1}A^{T}\left({\overset{-}{\beta }}_{D}\right)b. \end{array}$$

Based on the above discussion, we propose the following iterative linear least squares method.

**Step 1**

Choose the initial guess for **β**_*D*_^0^.

**Step 2**

Iteratively construct matrix **A**(**β**_*D*_^*s*^) and vector **b** by (28) and (29), respectively, and then solve the linear least squares problem:

(31) $$\begin{array}{c} J\left(\beta^{s+1}\right)={\left[A\left(\beta_{D}^{s}\right)\beta^{s+1}-b\right]}^{T}\left[A\left(\beta_{D}^{s}\right)\beta^{s+1}-b\right], \end{array}$$

which gives the solution

(32) $$\begin{array}{c} \beta^{s+1}={\left[A^{T}\left(\beta_{D}^{s}\right)A\left(\beta_{D}^{s}\right)\right]}^{-1}A^{T}\left(\beta_{D}^{s}\right)b \end{array}$$

until the stopping criterion is met, where **β** ^*s*^ = [**β**_*P*_^*sT*^,**β**_*N*_^*sT*^,**β**_*D*_^*sT*^]^*T*^ is the estimation of parameters **β** at step *s*.

From (31), if the estimation sequence **β** ^1^, **β** ^2^,… is converged to β*, the objective function (26) reaches its minimum value at β*. That is, β*is the estimation of parameters in model (22). 

There are several ways to set up a stopping criterion. In this paper the stopping criteria are chosen as

(33) $$\begin{array}{c} \frac{||\beta^{k}-\beta^{k-1}||}{||\beta^{k-1}||+1}\leq \varepsilon , \end{array}$$

where ||·|| is the Euclidean norm of the vector and *ε* is a preset small positive number, for example, 10^−5^. 

## 4. Application

**Figure 2:**
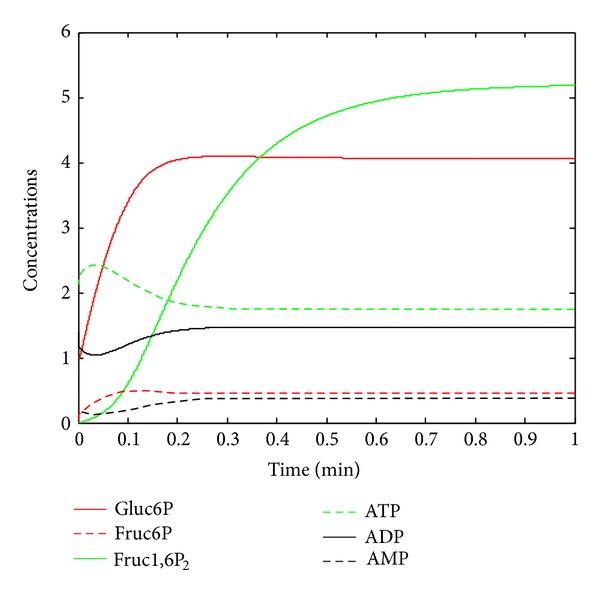
Trajectory of system (3).

To investigate the method developed in previous sections, this study generates artificial data from the dynamic metabolic system in the running example with the biochemically plausible parameter values [4] listed in column 2 of Table 1 and initial values: Gluc6P(0) = 1 mM, Fruc6P(0) = 0 mM, Fruc1,6P_2_(0) = 0 mM, ATP(0) = 2.1 mM, ADP(0) = 1.4 mM, and AMP (0) = 0.1 mM. The trajectory of this system is depicted in Figure 2. From Figure 2, the concentrations of all molecules except for Frucose-1,6-biphosphate reach their its steady states after about 0.1 minutes while Frucose-1,6-biphosphate after 0.5 minutes. Therefore, we do not use the data simulated after 0.5 minutes.

Although no noise is added to the artificial data in the simulation, noises are introduced in numerically calculating the derivatives by finite difference formulas. In general, the higher the sampling frequency and more data points are used, the more accurate the numerical derivatives are. On the other hand, we may not obtain data with the high frequency because of experimental limitations in practice. In this study, the sampling frequency is 100 data points per minute. In numerically calculating the concentration change rate at each time point from concentration *x*, we adopt the five-point central finite difference formula as follows:

(34) $$\begin{array}{c} \dot{x}\left(t_{n}\right)=\frac{1}{12\Delta t}\left[x\left(t_{n-2}\right)-8x\left(t_{n-1}\right)+8x\left(t_{n+1}\right)-x\left(t_{n+2}\right)\right]. \end{array}$$

The estimation accuracy of the proposed method is investigated in terms of relative estimation error which is defined as

(35) $$\begin{array}{c} \text{REE}=\frac{||\text{estimate\_value}-\text{true}\_\text{value}||}{||\text{true\_value}||}. \end{array}$$

As all parameters to be estimated are nonnegative, initial values are chosen as 0 or 1 in this study. The experimental results are listed in columns 3 and 4 in Table 1. From column 3 in Table 1, the estimated parameter values are very close to the corresponding true values. Actually the relative estimation errors calculated from (29) for all estimated parameters except for two are less than 1%. This indicates that the proposed method can accurately estimate the parameters in this system.

## 5. Conclusions and Future Work

In this study, we have first described a method to analyze the complexity of metabolic systems for parameter estimation, based on the stoichiometric matrix of the metabolic systems. As a result, the estimation of parameters in the metabolic systems has been reduced to the estimation of parameters in the improper rational functions or polynomial functions. Then we have developed an iterative linear least squares method for estimating parameters in the improper rational models. The results from its application to a metabolism system have shown that the proposed method can accurately estimate the parameters in metabolic systems.

We do not consider the noises in the data except those introduced by numerical derivatives in this study. One direction of future work is to investigate the influence of noises in the data to the estimation accuracy. In addition, low sampling frequency is expected, particularly for molecular biological systems as in practice measurements from them may be very expensive or it is impossible to sample measurements with high frequencies. Another direction of future work is to improve the estimation accuracy of the proposed method with low sampling frequencies.

## Figures and Tables

**Table 1:** The true value (from [4]), estimated value, and relative estimation errors.

Parameter name	True value	Estimated value	REE (%)
*V*_max⁡,2_ (mM·min^−1^)	50.2747	50.2447	0.0001
*K*_ATP,1_ (mM)	0.10	0.10000	0.0399
*k*_2_ (mM^−1^·min^−1^)	2.26	2.2599	0.0049
*V*_max⁡,3_^*f*^ (mM·min^−1^)	140.282	139.4917	0.5633
*V*_max⁡,3_^*r*^ (mM·min^−1^)	140.282	141.3623	0.7701
*K*_Gluc6P,3_ (mM)	0.80	0.7999	1.3884
*K*_Fruc6P,3_ (mM)	0.15	0.1499	0.0930
*V*_max⁡,4_ (mM·min^−1^)	44.7287	44.6664	0.1372
*K*_Fruc6P,4_ (mM^2^)	0.021	0.0206	1.8457
*k*	0.15	0.1526	1.7447
*k*_5_ (min^−1^)	6.04662	6.0466	0.0007
*k*_6_ (min^−1^)	68.48	68.4837	0.0054
*k*_7_ (min^−1^)	3.21	3.20797	0.0078
*k*_8*f*_ (min^−1^)	432.9	432.8408	0.0137
*k*_8*r*_ (min^−1^)	133.33	133.314	0.0120
